# Taxonomy of the *Proisotoma* complex. VI. Rediscovery of the genus *Bagnallella* Salmon, 1951 and epitoky in *Bagnallelladavidi* (Barra, 2001), comb. nov. from South Africa

**DOI:** 10.3897/zookeys.1072.71307

**Published:** 2021-11-23

**Authors:** Mikhail Potapov, Louis Deharveng, Charlene Janion-Scheepers

**Affiliations:** 1 Moscow State Pedagogical University, Kibalchicha str., 6, korp. 3, Moscow, 129278, Russia Moscow State Pedagogical University Moscow Russia; 2 Institut de Systématique, Evolution, Biodiversité, ISYEB-UMR 7205-CNRS, MNHN, UPMC, EPHE, Museum national d’Histoire naturelle, Sorbonne Universités, 45 rue Buffon, CP50, F-75005 Paris, France Sorbonne Universités Paris France; 3 University of Cape Town, Department of Biological Sciences, Rondebosch, 7701, Private Bag x3, South Africa University of Cape Town Cape Town South Africa; 4 Iziko Museums of South Africa, Cape Town, 8000, South Africa Iziko Museums of South Africa Cape Town South Africa

**Keywords:** Collembola, polymorphism, supermale

## Abstract

The genus *Bagnallella* Salmon is restored and given a diagnosis. It takes an intermediate position between *Proisotoma* Börner and *Cryptopygus* Willem complexes and is characterized by the separation of the two last abdominal segments (like in *Proisotoma*) and 3 and 5 s-chaetae on the fourth and fifth abdominal segments (like in *Cryptopygus* and its allies). A list of and key to species belonging to *Bagnallella* is given. *Bagnallellabiseta***comb. nov.**, *B.dubia***comb. nov.**, *B.sedecimoculata***comb. nov.**, and *B.tenella***comb. nov.** are commented and redescribed. Morphology of *Bagnallelladavidi* (Barra), **comb. nov.** is described from the specimens from South Africa. So far *B.davidi* appears to be a complex of forms differing in size of the furca and macrochaetae. Two types of strongly modified males were found and described. Antennae, ventral side of abdomen, posterior edge of abdominal tergites, and mandibles are affected with epitoky. The nature of the discovered strong polymorphism is unclear.

## Introduction

Knowledge on the Collembola of South Africa has increased significantly over the last decade, with most new species described from the Western Cape Province where the majority sampling has been made (Janion-Scheepers et al. 2015). From these collections, a rich diversity of Isotomidae has been discovered, including *Parisotoma* (Potapov et al. 2011) and *Cryptopygus* (Potapov et al. 2020). From these collections, we also recorded three known species (*P.davidi*, *P.tenella*, *P.sedecimoculata*), which resemble the genus *Cryptopygus* but have Abd. V and VI separated. Thus, these species cannot be attributed to any genus of the *Cryptopygus* complex but rather belong to the *Proisotoma* complex. This paper determines the position of these three species by recovering a genus erected in the past by Salmon (1951). Also, several other species mostly distributed in the Southern Hemisphere belong to this taxon. In addition, we describe an unusual polymorphism in *Proisotomadavidi* (Barra, 2001) which remains unsolved.

## Materials and methods

### Abbreviations

**A.B.** A. Bedos

**Abd. I–VI** abdominal segments I–VI

**Ant. I–IV** antennal segments I–IV

**bms** basal micro s-chaeta on antennal segments

**C.J.** C. Janion-Scheepers

**L.D.** L. Deharveng

**M** macrochaeta

**MNHN** Museum national d’Histoire naturelle

**ms**> micro s-chaeta(e) (= microsensillum(a) auct.)

**PAO** postantennal organ

**s-chaetae** macro s-chaeta or s-chaetae (= macrosensillum(a) or sensillum(a) auct.)

**SAMC**South African Museum, Cape Town

**Th. II–III** thoracic segments II and III

**Ti** tibiotarsus

## Redescription of the genus *Bagnallella* Salmon, 1951

### Bagnallella

Taxon classificationAnimaliaCollembolaCollembola

Salmon, 1951

FA6E65F7-465E-5545-A23A-15E1F95B8A1A

#### Type species.

*Folsomiasedecemoculata* Salmon, 1943

#### Diagnosis.

Anurophorinae with all abdominal segments clearly separated and a *Proisotoma*-like furca: manubrium with few anterior chaetae (1+1-3+3), dens slender, crenulated, with rather numerous anterior and posterior chaetae, mucro clearly set off from dens, with two or three teeth. 7+7-8+8 ocelli in known species. With simple or bifurcate maxillary palp and four sublobal hairs, two or four prelabral chaetae. Macro s-chaetae 22235 on Abd.I-V. Tergal macro s-chaetae on abdomen situated in front of p-row of chaetae. B-row of chaetae on Ti.1–2 complete (both B4 and B5 present). Ventral chaetae on Th.III present or absent. Sexual dimorphism present or absent.

#### Position of the genus in the subfamily Anurophorinae.

 To date an appropriate generic name did not exist for the small group species related to *Proisotoma* Börner, 1901 sensu lato which were discussed in the monograph of Potapov et al. (2006). This group, so-called “*Proisotomatenella*, *ripicola*, *biseta*”, consists of forms sharing characters such as: the three last abdominal segments separated, manubrium with anterior chaetae, four prelabral chaetae, and presence of three and five s-chaetae on Abd.IV and V, respectively.

Recently, one more species, *Proisotomasedecimoculata* (Salmon, 1943), became a probable candidate to belong to this group (Potapov and Janion-Scheepers 2017). This species was described by Salmon (1943) as *Folsomiasedecimoculata* and was afterwards proposed as a generotype for the new genus *Bagnallella* Salmon, 1951. *Bagnallella* was erected based on three last abdominal segments fused, bidentate mucro and eight ocelli. Later, *Bagnallella* was lost in the taxonomy of the subfamily and was mostly treated as a junior synonym of either *Folsomia* or *Proisotoma*. After the examination of the type specimen, it was discovered that the three last abdominal segments were actually separated (Potapov and Janion-Scheepers 2017). Here, we suggest restoring *Bagnallella* for the group of species mentioned above, rather than erecting a new generic name. Several other forms described under different generic names also fit to *Bagnallella* at lesser or larger degree of accuracy. For these species, the two *Bagnallella* key characters were mentioned in the associated descriptions or were seen by us, apart from three forms with unknown sensillar chaetotaxy. Nevertheless, we suppose the last ones (notated with question marks in the list of species of *Bagnallella* below) belong to the genus. Among these species, *Bagnallellasedecimoculata* is poorly described and so is not the best to be a generotype, but we prefer to keep a generic name already created by John Salmon.

**Figures 1–4.**
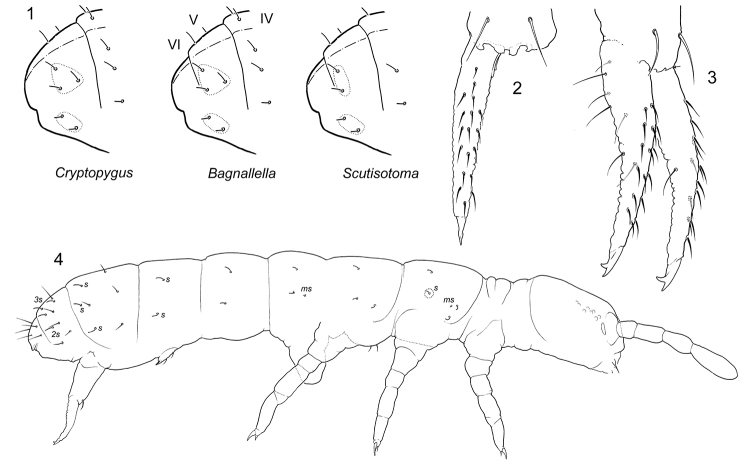
S-patterns of Abd. IV–VI in the genera *CryptopygusBagnallella* and *Scutisotoma* (**1**) *B.sedecimoculata* (**2**) and *B.dubia* (**3, 4**) **2, 3** furca, anterior view **4** macrochaetae and s and ms-chaetae on body. s = s-chaetae, ms = ms-chaetae.

The incertae sedis genus *Bagnallella* combines the characters of two large generic groups by the separation of its two last abdominal segments and the presence of three and five s-chaetae on Abd.IV and V respectively in characteristic position. The former character is a diagnostic feature of the *Proisotoma* Börner, 1901 complex, the latter indicates basic set of s-chaetae in *Cryptopygus* Willem, 1902 and related genera belonging to *Cryptopygus* complex (Potapov et al. 2006, 2013, 2020). The latter complex is characterized by the fusion of the two last abdominal segments. The genus *Bagnallella* takes a neatly intermediate position between *Cryptopygus* (*Cryptopygus* complex, Southern Hemisphere) and *Scutisotoma* (*Proisotoma* complex, Northern Hemisphere). The three genera share, apart from the characters of the subfamily, the presence of a furca, a mid-tergal position of macro s-chaetae on body tergites, and the absence of any specific apomorphy. The combinations of the two key characters mentioned above are shown in Figure 1. After the separation of the two last abdominal segments, we suggest treating *Bagnallella* in the *Proisotoma* complex.

#### Distribution and ecology of

***Bagnallella*.** The genus is distributed worldwide. More local species (*B.davidi*, *B.douglasi*, *B.mishai*, *B.biseta*, *B.koepckei*) are distributed in the Southern Hemisphere that indicates further relation to the “austral” genus *Cryptopygus*. Due to apparent ruderal *B.tenella* and pioneer *B.ripicola*, the genus also occurs in the Northern Hemisphere. The ecology of the former species is especially remarkable, as *B.tenella* is often recorded in mass abundances (Agrell 1939; Arle 1970; Neves and Mendonça 2016). We speculate that *B.davidi* has an unusual biology and ecology due to its morphological modifications (described below).

#### World list of the genus

##### Bagnallella

*Bagnallellabiseta* (Rapoport, 1963), comb. nov.

*Bagnallelladavidi* (Barra, 2001), comb. nov.

*Bagnallelladubia* (Deharveng, 1981), comb. nov.

*Bagnallelladouglasi* (Mendonça, Queiroz & Silveira, 2015), comb. nov.

? *Bagnallellakoepckei* (Winter, 1967), comb. nov.

*Bagnallellamishai* (Mendonça et Silveira, 2019), comb. nov.

? *Bagnallellanigromaculosa* (Folsom, 1932), comb. nov.

? *Bagnallellaparonai* (Börner, 1907), comb. nov.

*Bagnallellaripicola* (Linnaniemi, 1912), comb. nov.

*Bagnallellasedecimoculata* (Salmon, 1943), comb. nov.

*Bagnallellatenella* (Reuter, 1895), comb. nov.

#### Descriptions and remarks to species of the genus.

 Below we give the diagnosis, description, remarks, and distribution, with, if possible, ecological remarks of all species of *Bagnallella*. Some sections may be omitted if the associated species have good morphological descriptions in the literature.

### Bagnallella biseta

Taxon classificationAnimaliaCollembolaCollembola

(Rapoport, 1963) comb. nov.

65B31168-08FB-5E47-818C-063D4713BBAF

Proisotoma biseta Rapoport, 1963

#### Material examined.

 Adult male from Argentina under label “Tucuman, 19/4/59, entre maderas ...”, deposited in the Museum national d’Histoire naturelle (MNHN), Paris, France. This individual was obviously among the material the original description was based on by E.H. Rapoport.

#### Diagnosis.

 Maxillary palp bifurcate, four prelabral chaetae. Dens with ~50 anterior chaetae. Mucro bidentate. Anterior side of manubrium with 3+3 chaetae. 33/22235 s and 11/111 ms on body. Ventral chaetae absent on Th.III.

#### Description.

 Maxillary outer lobe with four sublobal hairs, maxillary palp bifurcate. Labral formula as 4/5,5,4. Guard chaeta e7 present on labium. Ant. III without bms and with five distal s (including one lateral), without additional s-chaetae. Th. I, II, and III without ventral chaetae. S-formula as 33/22235 (s), 11/111 (ms). Tibiotarsal tenent chaetae (1,1,1) clearly clavate. Tibiotarsi 1–2 with more than 24 chaetae. Ventral tube with 6+5 chaetae (in the adult male studied). Retinaculum with 4+4 teeth and two chaetae. Dens long and slender, with numerous crenulations, many (~50) anterior and 17 (in the adult male studied) posterior chaetae. Anterior side of manubrium with 3+3 chaetae. Mucro bidentate.

#### Discussion.

 Our redescription is based on one individual of E. Rapoport, and more material is needed to complete the understanding of *B.biseta*. The species obviously belongs to the genus *Bagnallella* by separation of two last abdominal segments and s-chaetotaxy of Abd.IV and V. After chaetotaxy of tibiotarsi, ventral tube, and dens, *B.biseta* appears to be a more polychaetotic species than its congeners. The close relation of *B.biseta* and *B.tenella* (sharing 3+3 manubrial chaetae) is doubtful due to difference in maxillary palp (bifurcate vs simple). The independence of *B.koepckei* and *B.paronai* from *B.biseta* call for further verification. *Bagnallellaparonai* is not included in the key due to the incomplete diagnosis.

#### Distribution.

 Argentina and Chile (see Mari Mutt and Bellinger 1990 for details).

### Bagnallella dubia

Taxon classificationAnimaliaCollembolaCollembola

(Deharveng, 1981) comb. nov.

7F0F127F-D593-50F9-8D40-8961E0A269E3

Figures 3 , 4

Cryptopygus dubius Deharveng, 1981

#### Material examined.

 New Zealand, South Island, Central Otago, Pisa Range and Old Man’s Range, high alpine zone, different sites, 17.02.2014, M. Minor leg.

#### Diagnosis.

 Maxillary palp bifurcate, two prelabral chaetae. Dens with 12–16 anterior chaetae. Mucro bidentate. Anterior side of manubrium with 1+1 chaetae. 33/22235 s and 10/100 ms on body. 2+2 ventral chaetae on Th.III.

#### Description.

 Colour grey. Cuticle, ocelli, outer mouth parts, and antennae as in *B.sedecimoculata*. PAO as long as 0.8–0.9 Ant. I and as 1.4–1.5 as long as Claw III. Ventral side of head with 4+4 postlabial chaetae. Th.III with 2+2 ventral axial chaetae.

Macrochaetae weakly differentiated, medial ones on Abd.V about as long as 0.4–0.5 of tergal midline. S-chaetae weakly differentiated. S-formula as 43/22235 (s), 10/100 (*ms*) (Fig. 4). S-chaetae on Abd.I–III in mid-tergal position. Tibiotarsi 1–2 with 21 chaetae, Tibiotarsi 3 with few additional chaetae. Tibiotarsal tenent chaetae not developed. Ventral tube with 4+4 laterodistal and usually with five posterior chaetae. Retinaculum with 4+4 teeth and two chaetae. Anterior furcal subcoxae with 13–15 chaetae, posterior ones with 7–9. Anterior side of manubrium with 1+1 distal chaetae. Dens with 12–16 anterior chaetae, posterior side of dens with crenulation and seven chaetae (Fig. 3). Mucro bidentate. Ratio of manubrium : dens : mucro = 6.0–6.7 : 5.0–6.0 : 1. Males present, with two thin spurs on Tibiotarsi I.

#### Discussion.

 This species was named after its dubious position in generic system of *Proisotoma*/*Cryptopygus* (Deharveng 1981). It resembles *B.sedecimoculata* (see the Discussion below) and apparently belongs to the genus *Bagnallella* by separation of two last abdominal segments and s-chaetotaxy of Abd.IV and V. Our specimens from New Zealand match the first description.

#### Distribution.

*Bagnallelladubia* was described from Marion Island and recorded in Macquarie Island (Greenslade and Wise 1986) and alpine sites of New Zealand (Babenko and Minor 2015). The species is possibly widely distributed in cold sites of high altitudes of the Southern Hemisphere. Its occurrence in Australia (Greenslade 2006) needs to be verified.

### Bagnallella douglasi

Taxon classificationAnimaliaCollembolaCollembola

(Mendonca, Queiroz & Silveira, 2015) comb. nov.

0639924F-46AD-5937-8DDB-BC24B98846E2

Proisotoma douglasi Mendonca, Queiroz & Silveira, 2015.

#### Discussion.

 The species can be attributed to *Bagnallella* by the two key characters of the genus. It is characterized by 33/22235 s, 11/111 ms, bifurcate maxillary palp, long polychaetotic dens (34-35/14), and bidentate mucro. The presence of seven ocelli and 2+2 chaetae on anterior side of manubrium are two unique characters among members of the genus.

#### Distribution.

 This species is currently only known from SE Brazil.

### Bagnallella mishai

Taxon classificationAnimaliaCollembolaCollembola

(Mendonca & Silveira, 2019) comb. nov.

BA7B4AC4-AE5D-5465-8A9A-392254AB49C6

Scutisotoma misha Mendonca & Silveira, 2019

#### Discussion.

 It is an easily recognizable species by 43/22235 s, 11/111 ms, simple maxillary palp, dens (15-16/12-13), and tridentate mucro.

#### Distribution.

 One locality in southeastern Brazil.

### Bagnallella ripicola

Taxon classificationAnimaliaCollembolaCollembola

(Linnaniemi, 1912) comb. nov.

10A2360F-8B8A-5744-8C21-FD4084B80CFE

Proisotoma ripicola Linnaniemi, 1912

#### Diagnosis.

 Maxillary palp bifurcate, four prelabral chaetae. Ant. I with many additional chaetae. Dens long and slender, with 20–30 anterior chaetae or more. Mucro bidentate. Anterior side of manubrium with 1+1 chaetae. 33/22235 s and 11/111 ms on body. Lateral s-chaetae on Abd.IV shifted to ventral side. Without ventral chaetae on Th.III.

#### Discussion.

 The full redescription is given by Fjellberg (2007).

#### Distribution and ecology.

 Europe. It prefers sandy places along the edge of water.

### Bagnallella sedecimoculata

Taxon classificationAnimaliaCollembolaCollembola

(Salmon, 1943)

758841A8-3C8C-512D-BB51-61DBE84910CD

Figure 2

Folsomia sedecimoculata Salmon, 1943
Holotoma sedecimoculata (Salmon, 1943)
Proisotoma sedecimoculata (Salmon, 1943)

#### Material examined.

 South Africa, Western Cape, Stellenbosch, Jonkershoek Nature Reserve, canyon to waterfall, SAF-086, 34.005570°S, 18.992067°E, 15/03/2008, forest litter, Berlese, L.D. and A.B. leg.; Somerset, Helderberg, SAF-107, SAF-109, SAF-116, 34.040883°S, 18.873649°E, alt. 600 m, 04/03/2009, native forest litter, L.D. and A.B. leg.; Cape Town, Wynberg, Table Mountain, second collapse, SAF-141, 33.987637°S, 18.405750°N, alt. 725 m, 10/03/2009, native forest litter, L.D. and A.B. leg.; Constantia, Silvermine, in a small forest patch above Tokai, SAF-235, 34.038273°S, 18.395478°E, alt. 390 m, 06/11/2010, dead wood, D. Porco leg.; Kalk Bay, Echo Valley, Spes Bona forest, SAF-555, 01/03/2019, Afromontane forest, moss on rock, L.D. and A.B. leg.

New Zealand. NZL-049, Rotoiti: Lakes Rototongata and Rotoatua, 08/01/1996, primary forest, litter, L.D. and A.B. leg.

Australia. Victoria, July 2010, University Ballarat, St. Helens, 37.629979°S, 143.890801°E, *Eucalyptus* plantation, moss, P. Greenslade. leg.

Macquarie Island, Bauer Bay, 54.5549°S, 158.8760°E, April 2016, Turf sample, Berlese extraction, L. Phillips leg.

#### Diagnosis.

 Maxillary palp bifurcate, two prelabral chaetae. Dens slender, with 16–20 anterior chaetae. Mucro bidentate. Anterior side of manubrium with 1+1 chaetae. 33/22235 s and 10/100 ms on body. Without ventral chaetae on Th.III.

#### Description.

 Colour grey. Cuticle outwardly smooth. 8+8 ocelli, G and H smaller. PAO about as long as 0.8 Ant. I and as 1.1–1.3 Claw III. Maxillary outer lobe with four sublobal hairs and bifurcate maxillary palp. Labral formula as 2/554. Labium full set of guards (e7 present), three proximal and four basomedian chaetae. Ventral side of head with 4–5+4–5 postlabial chaetae. 11 chaetae on Ant.I, with three basal micro s-chaetae (*bms*), of which one *bms* large, and two ventral s-chaetae (s), Ant.II with three *bms* and one laterodistal s, Ant.III with one *bms* and six distal *s* (including two lateral). Thorax without ventral axial chaetae.

Macrochaetae weakly differentiated, medial ones on Abd.V as long as 0.4–0.5 of tergal midline. S-chaetae weakly differentiated. S-formula as 43/22235 (s), 10/100 (*ms*). S-chaetae on Abd.I–III in mid-tergal position. General pattern of chaetotaxy as in *B.dubia* (Fig. 4). Ti.1–2 with 21 chaetae, Ti.3 with few additional chaetae. Tibiotarsal tenent chaetae not developed. Ventral tube with 4+4 laterodistal and usually with six posterior chaetae (four in a transversal row). Retinaculum with 4+4 teeth and two chaetae. Anterior furcal subcoxae with 11–15, posterior ones with 7–9 chaetae. Anterior side of manubrium with 1+1 distal chaetae (Fig. 2). Dens with 17–19(16–20) anterior chaetae, posterior side of dens with crenulation and seven chaetae (Fig. 2). Mucro bidentate. Ratio of manubrium : dens : mucro = 5.3–6.3 : 5.9–6.7 : 1.

#### Discussion.

*Bagnallellasedecimoculata* was described from New Zealand and was designated as type species for the genus *Bagnallella* (see the discussion to the genus above). The type specimen from New Zealand was studied (Potapov and Janion-Scheepers 2017), although only one generic character (separation of two abdominal segments) was proven. The redescription given above is based mostly on the South African material which looks conspecific to one individual in hand from New Zealand (L.D. and A.B. leg.). *Bagnallellasedecimoculata* resembles *B.dubia* and differs by ventral chaetae on Th.III (absent vs present) and a few more anterior chaetae on dens (16–20 vs 12–16). The latter character is not stable enough to separate the two species.

#### Distribution.

 Probably widely distributed. So far with scattered records in the South Hemisphere (New Zealand, Australia, South Africa, Macquarie Island).

### Bagnallella tenella

Taxon classificationAnimaliaCollembolaCollembola

(Reuter, 1895) comb. nov.

06F19A81-B54A-58ED-8836-5E6555B4317F

Figures 5–8

Isotoma tenella Reuter, 1895
Proisotoma tenella (Reuter, 1895)
Proisotoma simplex Folsom, 1937
Proisotoma alba Yosii, 1939

#### Material examined.

 South Africa, Western Cape, Haarwegskloof, Swellendam, 34.3425°S, 20.3167°E, 18.vii.2017, litter trap (R17) with *Dicerothamnusrhinocerotis* litter, O. Cowan leg.; Haarwegskloof, Swellendam, 34.3444°S, 20.3225°E, 18.vii.2017, litter trap (A17) with *Medicagosativa* litter, O. Cowan leg.; Eastern Cape, Baviaanskloof, 33.7311°S, 23.9655°E, 24.iv.2013, BAV_F_49, A. Liu leg.; Free State Province, Bankfontein Farm, 30.0567°S, 24.8942°E, 24.iv.2019, Berlese-Tullgren, tree leaf litter, H. Badenhorst leg.

**Figures 5–8.**
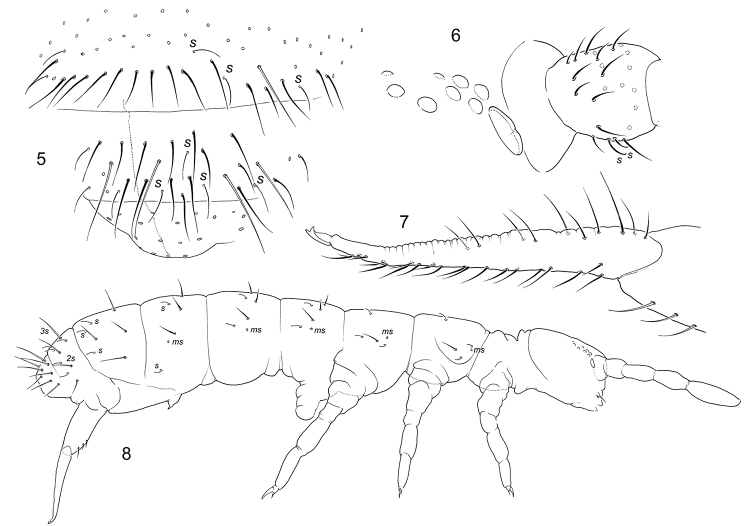
*Bagnallellatenella***5** chaetotaxy of posterior part of Abd. IV, Abd.V and VI **6** ocelli, PAO, and Ant. I **7** furca, lateral view **8** macrochaetae and s and ms-chaetae on body. s = s-chaetae, ms = ms-chaetae.

Cuba, Cienfuegos Province, 1984, J. Banasco-Almenteros leg.

Indonesia, Lombok Island, vic. Toko Nusa Sari, marine beech, 8.7411° S, 116.0011° E, 4.IV.2017, V. Makarov leg.

Brazil, Espirito Santo, Domingos Martins, Arace, 4.VII.2000, M. Culik leg.

#### Diagnosis.

 Maxillary palp simple. Four prelabral chaetae. Dens slender, with ca 20–30 anterior chaetae. Mucro bidentate. Anterior side of manubrium with 3+3 chaetae. 33/22235 s and 11/111 ms on body. No ventral chaetae on Th.III.

#### Description.

 Colour grey of different intensity. Cuticle outwardly smooth. 8+8 ocelli, G and H smaller. PAO (Fig. 6) about half as long as width of Ant.I and 0.8–0.9 as long as Claw 3. Maxillary outer lobe with four sublobal hairs and simple maxillary palp. Labral formula as 4/554. Labium without guards e7, with three proximal and four basomedian chaetae. Ventral side of head with 4-5+4-5 postlabial chaetae. With several additional chaetae on Ant.I. Ant.I with three basal micro s-chaetae (*bms*), one dorsal large, Ant.II with three *bms*, Ant. III without *bms*. Thorax without ventral axial chaetae. Macrochaetae rather long, differentiated (11/3334), medial ones on Abd.V about as long as tergal midline. S-chaetae on tergites slightly shorter than ordinary chaetae. S-formula as 33/22235 (s), 11/111 (*ms*) (Figs 5, 8). S-chaetae subequal, on Abd.I–III in mid-tergal position. Micro s-chaetae on Abd.I–II in front of lateral s-chaetae, on Abd.III between medial and lateral s-chaetae (Fig. 8). Tibiotarsal tenent chaetae (1,1,1) often present, weakly clavate (see Discussion). Retinaculum with 4+4 teeth and 1–2 chaetae. Furca long. Anterior side of manubrium with 3+3 chaetae arranged in two lines (Fig. 7). Dens with more than 20 anterior chaetae, posterior side of dens with crenulation and 7–10 chaetae (Fig. 7). Mucro bidentate. Ratio of manubrium : dens : mucro = 7–8 : 9 : 1.

#### Discussion.

 The species belongs to the genus *Bagnallella* by having two last abdominal segments separated and three and five s-chaetae on Abd. IV and V. It shares 3+3 anterior chaetae on manubrium with *B.biseta*, *B.koepckei*, and *B.paronai* from South America from which it differs by fewer chaetae on dens.

Number of posterior chaetae on dens, clavate tibiotarsal chaetae, and chaetae on retinaculum vary. We treat all this variation within one species, but further study is needed. The independence of *Proisotomanigromaculosa* (Hawaiian Islands) is doubtful.

Although we have no material from Europe, Stach’s (1947) concept of *P.tenella* based on the specimens from Poland is accepted by us (for details see Potapov 2001; Fjellberg 2007). Our tropical material fits Stach’s (1947) descriptions.

#### Distribution and ecology.

 Widely distributed cosmopolitan species. Common in tropics. In higher latitudes only in protected soils.

##### Description of *Bagnallelladavidi* and its forms in South Africa

### Bagnallella davidi

Taxon classificationAnimaliaCollembolaCollembola

(Barra, 2001) comb. nov.

A32B9BE7-D66F-5818-AB4D-BE40E47FEB79

Figures 9–14 , 15–19 , 20 , 21–24

Proisotoma davidi Barra, 2001

#### Material examined

**. Typical form**: South Africa, Free State Province, Bankfontein Farm, 30.0567°S, 24.8942°E, 24.iv.2019, Berlese-Tullgren: tree leaf litter, H. Badenhorst leg.; South Africa, Western Cape, Haarwegskloof, Swellendam, 34.3422°S, 20.3169°E, 18.vii.2017, litter trap (G18) with *Pentameriseriostoma* litter, O. Cowan leg., South Africa, SAF 583 (11.m CJ SWB); Prince Albert: Swartberg North: road to Swartberg Pass, 11/03/2019, meadow, moss, C.J. leg.

**Short-haired form**: South Africa, Western Cape, Haarwegskloof, Swellendam, 34.3534°S, 20.3042°E, 18.vii.2017, litter trap (A4) with *Medicagosativa* litter, O. Cowan leg.; Cederberg Wilderness area, Litter trap CED588; South Arica, Western Cape, Cederberg Wilderness area, Litter trap CED394; Jonkershoek Nature Reserve, 33.9891°S, 18.9575°E, 05.ix.2011, Litter trap (J4, 124); Jonkershoek Nature Reserve, 33.9891°S, 18.9575°E, 30.vii.2009, Litter trap, C.J. leg.; J2, 32.1; Landdroskop, Jan. 2012, H. Basson leg.; Prince Albert, Swartberg North, Swartberg crest, 12/03/2019, SAF-612, SAF-618, meadow, litter and soil, L.D., C.J. and A.B. leg.

**Intermediate form**: South Africa, Western Cape, Haarwegskloof, Swellendam, 34.3345°S, 20.3187°E, 18.vii.2017, litter trap (R24) with *Dicerothamnusrhinocerotis* litter, O. Cowan leg.; Prince Albert, Swartberg North, Swartberg crest, SAF-612, 12/03/2019, meadow, litter, L.D., C.J. and A.B. leg.; Prince Albert, Swartberg North, Swartberg crest, SAF-618, 12/03/2019, meadow, soil, L.D., C.J. and A.B. leg.; Prince Albert, Swartberg North, road to Swartberg Pass, SAF-601, 12/03/2019, meadow, litter and soil, L.D., C.J. and A.B. leg.; Prince Albert, Swartberg North, Swartberg crest, SAF-614, 12/03/2019, moss on rock, Berlese, L.D., C.J. and A.B. leg.

“**Clasping supermales**”: SAF-601, South Africa: Western Cape: Prince Albert: Swartberg North: road to Swartberg Pass, 12/03/2019, meadow, litter and soil, L.D., C.J. and A.B. leg.

“**Spiny supermales**”: SAF-554; South Africa: Western Cape: Kalk Bay: Echo Valley: Spes Bona forest, 01/03/2019, Afromontane forest, moss and lichen on rock, L.D. and A.B. leg.

#### Diagnosis.

**Figures 9–14.**
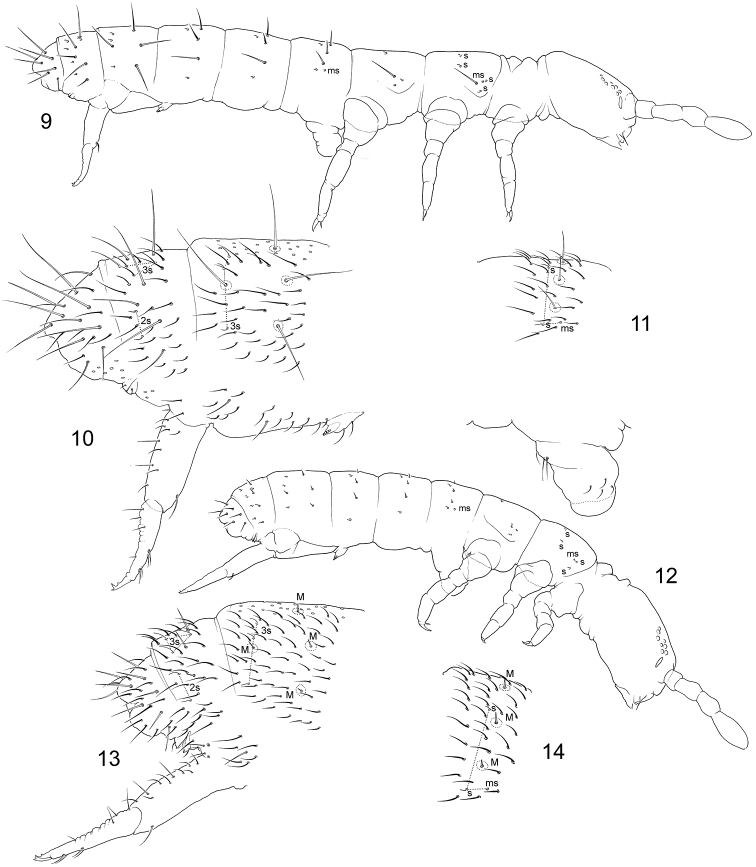
*Bagnallelladavidi*, normal long-haired (**9–11**) and short-haired form (**12–14**) **9, 12** macrochaetae and s and ms-chaetae on body **10, 13** posterior part of abdomen **11, 14**Abd.I. s = s-chaetae, ms = ms-chaetae.

 3+3 postlabial chaetae. Maxillary palp simple. Dens with four anterior and four posterior chaetae. Mucro tridentate, teeth arranged in a line. Anterior side of manubrium with 1+1 chaetae. 43/22235 s and 10/100 ms on body (Figs 9–11). No ventral chaetae on Th.III. Typical form of species with long macrochaetae (Fig. 10).

#### Description.

 Maxillary outer lobe with four sublobal hairs, maxillary palp simple. Labral formula as 4/5,5,4. Labium with five usual papillae (–Е) and labial formula A1B4C0D4E6, guard chaeta e7 absent, three proximal and four basomedian chaetae. Ventral side of head with 3+3 chaetae. PAO shorter than Ant.I width (0.6–0.8). Ant. I with 11 common chaetae, two ventral s-chaetae (s) and three bms, of which one dorsal long, chaeta-like (this ms was calculated together with common chaetae in first description, 12 at whole); Ant. II with three bms and one latero-distal s; Ant. III with one bms and with six distal s (including two lateral), without additional s-chaetae. Organite pin-like, not very small. Empodial appendage about half as long as Claw. Anterior and posterior furcal subcoxae with 9–14 and 7–8 chaetae, respectively. Male spurs on tibiotarsi 3 thin, stick-like. Th. I–III without ventral chaetae. Ratio manubrium : dens : mucro as 4.4-5.0 : 3.3-3.8 : 1; dens : Claw as 3.3–3.6 (for the typical form).

#### Discussion.

*Bagnallelladavidi* is a rather peculiar species of the genus due to few chaetae on dens (vs many more chaetae both on anterior and posterior sides), tridentate mucro (shared with *B.mishai* only), and 3+3 postlabial chaetae (vs 4+4 or more in other species). The first description is almost complete and, therefore, we made very few additions concerning mouth parts. The species exhibits high variation in length of macrochaetae and show different modifications of males. All the forms (described below) can indicate either high plasticity of a single species or the complex of separate although closely related species, calling for further morphological, biological, ecological, and molecular investigations.

#### Distribution.

 Eastern Cape, Amatola Mountains (type location) and widely in the Western Cape and Free State (our material) provinces of South Africa.

##### Polymorphism of *Bagnallelladavidi*

1. “Typical “ form (Figs 9–11). B. davidi was described in this form (Barra 2001). Macrochaetae on body segments are long. Ratios: Mac on Abd.V as long as 0.7–1.0 of tergal midline. Mac : Abd.V width = 0.7–1.0; Mac : mucro = 3.3–4.1; Mac : dens = 0.8–1.1 (Fig. 9). In Proisotoma complex, so long macrochaetae is a unique character among species of Bagnallella and sometimes occur in the genera Weberacantha Christiansen, Narynia Martynova and Folsomides Stach. This form was found in juvenile and fully adult specimens, both in females and males.
2. “Short-haired” form (Figs 12–14). Macrochaetae are short, shorter than common chaetae on most abdominal segments. Ratios: Mac : Abd.V Mac on Abd.V as long 0.2–0.3 of tergal midline; Mac : mucro = 0.9–1.4; Mac : dens 0.2–0.3. In spite of their small size, macrochaetae are erect and stiff and so well recognized indicating their possible ecomorphic nature although the integument and mouthparts are not modified. Head and furca appear to be relatively larger than in typical form. Ratio manubrium : dens : mucro as 5.1–6.6 : 4.1–5.8 : 1; dens : Claw as 3.3–4.5. All other significant characters (s-chaetotaxy, mouth parts, chaetotaxy of extremities) are as in typical form. All instars and both sexes can belong to this form.

**Figures 15–19.**
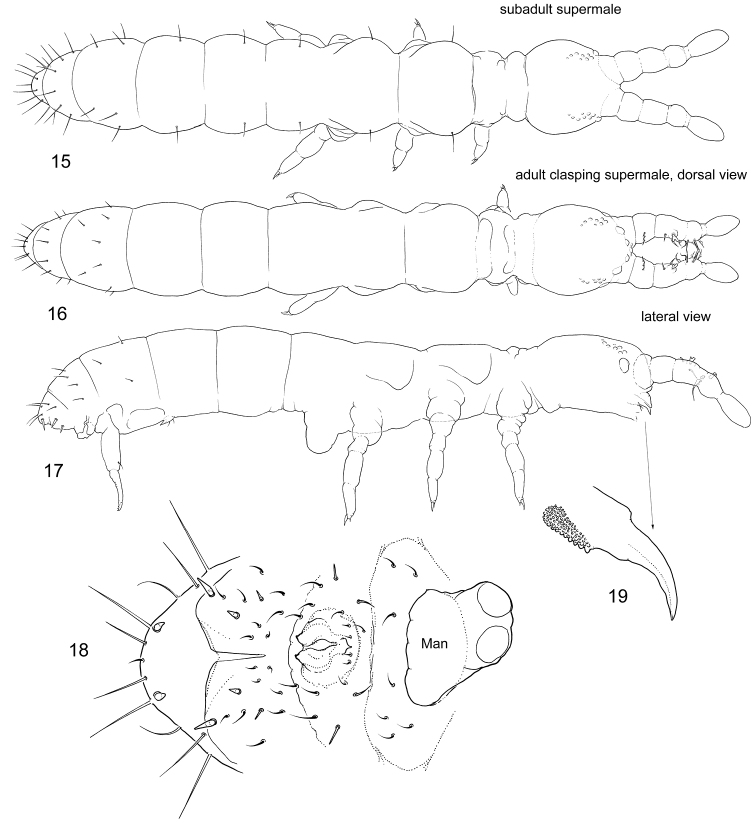
*Bagnallelladavidi*, the “clasping supermale” **15** subadult individual, dorsal view **16, 17** adult, dorsal **16** and lateral **17** views **18** posterior part of abdomen in adult male, ventral view **19** mandible. Man = Manubrium.

We also found individuals with middle-sized macrochaetae (as in Fig. 15), which is on Abd.V twice as long as mucro (vs 3.3–4.1 in typical form and 0.9–1.4 in short-haired form) and half as long as dens (vs subequal to dens in typical form and 0.2–0.3 as dens in short-haired form).

Each studied population consists of only one of the forms, and we have not found a continuous range of macrochaetae variability, apart from short-haired clasping adult supermales occurring in “normal” populations.

**Figures 20.**
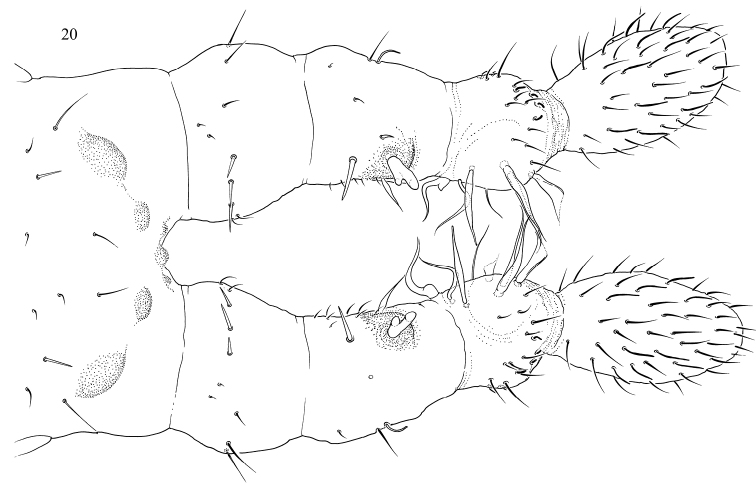
*Bagnallelladavidi*, anterior part of head and antennae in adult “clasping supermale”.

1. “Clasping supermales” (Figs 15–20). Ant.I–III expanded and partly fused. Antennae joints probably lost mobility. Inner side of Ant.II and III is armed with thickened, flame-shaped, and bifurcate chaetae which probably form a clasping organ. Front of head have chitinized tubercles. Anal valves are armed with spines. Mandibles without apical teeth. Macrochaetae short. Subadult clasping supermales, i.e., males without fully developed genital plate and without developed ejaculatory duct, have also expanded antennae although without modified chaetae on inner their side. They show normal (longer than on adult supermales) macrochaetae and normal mandibles and have no spines on anal valves (Fig. 15–20). The females of the same population belong to the form with middle-sized macrochaetae.
2. “Spiny supermales” (Figs 23–25). One of the males has a row of spiny p-chaetae on Abd.IV and strong thickened macrochaetae on lateral parts of Abd.VI. Other macrochaetae on the body are weakly developed. Common chaetae on dorsum of Abd.IV–VI curved at apex (Fig. 25). Mandibles without apical teeth and molar plate (Fig. 24). Outer mouth parts (labrum, maxillary outer lobe, and labium) not fully developed.

**Figures 21–25.**
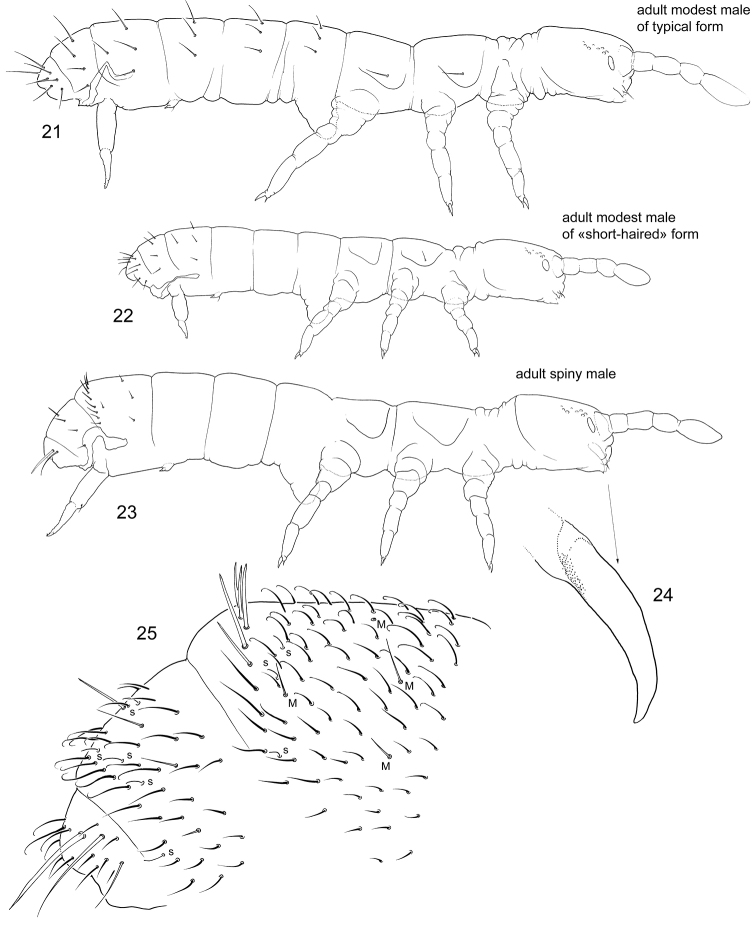
*Bagnallelladavidi* unmodified (**21, 22**) and “spiny supermale” (**23–25**) **21–23** lateral view **24** mandible **25** posterior part of abdomen, lateral view. s = s-chaetae, M = macrochaetae.

Unmodified males are much more frequent than the two male forms described above. In most populations, only unmodified males are known. They can show all possible length of macrochaetae and belong to associated forms (Figs 21, 22).

### Key to known species of *Bagnallella*^1^

**Table:** 

1	Mucro tridentate (Fig. 10)	**2**
–	Mucro bidentate (Fig. 3)	**3**
2	Dens with 4 anterior and 4 posterior chaetae (Fig. 10)	***B.davidi* (Barra), South Africa**
–	Dens with 15–16 anterior and 12–13 posterior chaetae	***B.mishai* (Mendonça & Silveira), Brazil**
3	Manubrium with 1+1 anterior chaetae (Fig. 3)	**4**
–	Manubrium with 2–3+2–3 anterior chaetae (Fig. 7)	**6**
4	Dens with more than 20 anterior and 15 posterior chaetae. 4 prelabral chaetae	***B.ripicola* (Linnaniemi), Europe**
–	Dens with fewer than 17 anterior and 8 posterior chaetae (Figs 2, 3). 2 prelabral chaetae	**5**
5	Ventrum of Th.III with chaetae	***B.dubia* (Deharveng), sub-Antarctic**
–	Ventrum of Th.III without chaetae	***B.sedecimoculata* (Salmon), New Zealand**
6	Manubrium with 2+2 anterior chaetae, 7 ocelli	***B.douglasi* (Mendonça, Queiroz & Silveira), Brazil**
–	Manubrium with 3+3 anterior chaetae, 8 ocelli	**7**
7	Dens with more than 40 anterior chaetae	***B.biseta* (Rapoport), Argentina**, ***B.koepckei* (Winter), Peru**^2^
–	Dens with fewer than 30 anterior chaetae (Fig. 7)	***B.tenella* (Reuter), cosmopolitan**, ***B.nigromaculosa* (Folsom), Hawaii Is**^2^

## Discussion

If present, the clasping organ of Collembola is formed by two symmetrical complexes associated with, respectively, left and right antennae, or, more rarely, other limbs. Paired “clasps” are evolutionary formed in different taxa, for example, in the family Sminthurididae, *Vertagopusreuteri* (Schött, 1893), *Rhodanellaminos* Denis, 1928, *Seiraraptora* (Zeppelini & Bellini, 2006) (Delamare Deboutteville et al. 1969; Betsch 1980; Fjellberg 1982; Bellini et al. 2009). In “clasping supermales” of *Bagnallelladavidi*, all modified chaetae (curved spines, flame-shaped, flattened, and bifurcate) are found on the inner side of the antennae, forming a unique type of clasping organ. This allows males to grasp females between the two antennae at the axial region. The only possible similar case was described in *Vertagopuspseudocinereus* Fjellberg, 1975, which clasps the female with right and left antennae. This species has curved and serrated chaetae on antennae, without any strong modifications (Goloschapova et al. 2006: fig. 2). We can assume that males of *B.davidi* uses this unpaired “clasp” (Fig. 20) in a similar manner, for clasping onto the female.

The function of the spiny row in “spiny supermales” of *B.davidi* is more difficult to explain. The armature of the posterior row on Abd.IV somewhat resembles backward-shifting macrochaetae in males of *Scutisotomaacorrelata* Potapov, Babenko & Fjellberg, 2006, while strong lateral macrochaetae on Abd. VI indicate some similarity to *Ephemerotomahuadongensis* (Chen, 1985), which shows similar armature on both Abd. V and VI (described by Potapov et al. 2015, 2020, respectively).

## Supplementary Material

XML Treatment for Bagnallella

XML Treatment for Bagnallella biseta

XML Treatment for Bagnallella dubia

XML Treatment for Bagnallella douglasi

XML Treatment for Bagnallella mishai

XML Treatment for Bagnallella ripicola

XML Treatment for Bagnallella sedecimoculata

XML Treatment for Bagnallella tenella

XML Treatment for Bagnallella davidi

## Footnotes

Some known species of the Proisotoma complex with insufficient description may belong to the genus Bagnallella.

At present Bagnallella biseta cannot be reliably discriminated from Proisotoma koepckei based on the existing descriptions. A similar problem exists with B. tenella and P. nigromaculosa.
